# A Comparative Proteomic Analysis of the Simple Amino Acid Repeat Distributions in *Plasmodia* Reveals Lineage Specific Amino Acid Selection

**DOI:** 10.1371/journal.pone.0006231

**Published:** 2009-07-14

**Authors:** Andrew R. Dalby

**Affiliations:** Department of Statistics, University of Oxford, Oxford, United Kingdom; University of California San Diego, United States of America

## Abstract

**Background:**

Microsatellites have been used extensively in the field of comparative genomics. By studying microsatellites in coding regions we have a simple model of how genotypic changes undergo selection as they are directly expressed in the phenotype as altered proteins. The simplest of these tandem repeats in coding regions are the tri-nucleotide repeats which produce a repeat of a single amino acid when translated into proteins. Tri-nucleotide repeats are often disease associated, and are also known to be unstable to both expansion and contraction. This makes them sensitive markers for studying proteome evolution, in closely related species.

**Results:**

The evolutionary history of the family of malarial causing parasites *Plasmodia* is complex because of the life-cycle of the organism, where it interacts with a number of different hosts and goes through a series of tissue specific stages. This study shows that the divergence between the primate and rodent malarial parasites has resulted in a lineage specific change in the simple amino acid repeat distribution that is correlated to A–T content. The paper also shows that this altered use of amino acids in SAARs is consistent with the repeat distributions being under selective pressure.

**Conclusions:**

The study shows that simple amino acid repeat distributions can be used to group related species and to examine their phylogenetic relationships. This study also shows that an outgroup species with a similar A–T content can be distinguished based only on the amino acid usage in repeats, and suggest that this might be a useful feature for proteome clustering. The lineage specific use of amino acids in repeat regions suggests that comparative studies of SAAR distributions between proteomes gives an insight into the mechanisms of expansion and the selective pressures acting on the organism.

## Introduction

Malaria is the main threat to human health in developing countries. Each year there are between 400 and 900 million cases in children under 5 in Africa alone and 2.7 million people die of the disease [1]. With global warming there is a threat that the disease will become more wide-spread and return to Europe and North America. Most deaths are caused by the *Plasmodium falciparum* which has also developed resistance to many existing anti-malarial therapies.

A deeper understanding of the evolution of the malarial parasites would help in our understanding of drug activity and the development of resistance and may also provide potential new therapeutic targets. Understanding the evolution of pathogenicity and the relationship between vectors and parasites is also important in trying to target new treatments for the disease. The most effective treatment for the developing world would be a vaccine. By building a deeper understanding of the parasitic life-cycle and its host-specific proteome regulation and evolution we can identify potential leads for vaccine development [2].

The first malarial genome sequenced was that of *Plasmodium falciparum* 3D7 and since then it has been the object of intense study [3]. Now we also have the complete annotated genomes of *Plasmodium vivax* and the primate malarial parasite *Plasmodium knowlesi*. The rodent malarial parasites *Plasmodium chabaudi, Plasmodium berghei* and *Plasmodium yoelii* have also been sequenced and their genomes are available from the Sanger Centre and The J. Craig Venter Institute [4]–[6]. The genome of the chimpanzee malaria *Plasmodium reichenowi* is also available as unassembled contigs from shotgun sequencing, but currently a complete proteome is not available. Complete proteomes are available from the Integr8 database for *P. falciparum* (3D7), *P. berghei* (Anka strain), *P. chabaudi*, *P.knowlesi, P.vivax* and *P yoelii yoelii*.

The sequencing of multiple *Plasmodium* genomes allows the study of parasite evolution using comparative genomics. Comparing entire genomes is a challenging problem and so efforts are focused on particular features of the genomes or on particular coding sequences [7], [8]. Studies on *P.falciparum* have also used the sequencing of multiple isolates to look at the fine-scale structure of genome evolution and population genetics [9]–[11]. This allows the process of recombination within the parasite genome to be studied. One set of markers that are used to evaluate the similarity or differences between members of a population are micro-satellites because of their rapid evolution [12]. Micro-satellites can be divided into two groups, those in coding and those in non-coding regions. The two types exhibit very different properties. Repeats within coding regions are tri-nucleotide repeats as other repeat sizes bring about a frameshift which would result in the translation of a completely different protein and so they expand by particular mechanisms that are cell type dependent [13]–[15]. In non-coding regions there are less constraints and di-nucleotide repeats are more common [16].

Similarly comparisons can be made at the proteomic level which gives an alternative set of features for comparison. The simplest of these are the simple amino acid repeats (SAARs), which correspond to the genomic tri-nucleotide repeats in coding regions. These are long homogeneous runs of single amino acids within a protein sequence. SAARs have been found for all twenty amino acids in all three domains (Eukaryotes, Archaea and Bacteria), although they are not found in all classes of proteins and they are generally absent from metabolic enzymes and heat shock proteins [17]–[19]. SAARs account for between 12–14% of the amino acid content of any particular proteome and a repeat of 20 amino acids length can be found in most organisms [18].

In humans several genetic diseases are associated with SAARs and the extension of the repeat by tri-nucleotide expansion. The most well characterized example is Huntington's Chorea where there is a long glutamine repeat which undergoes tri-nucleotide expansion. Proteins that contain one tri-nucleotide expansion often contain others and so they often contain multiple SAARs. For example Huntingtin contains Q_23_, P_11_, P_10_, E_5_ and E_6_ repeats [20].

Previous research has analyzed the low complexity regions in *Plasmodia* and also identified what are termed intrinsically unordered regions (IURs) [9], [21]–[23]. Intrinsically unordered regions are regions of sequence that do not form secondary structural elements that have been shown to evolve by repeat expansion [24]. As well as being tandem repeats and microsatellites SAARs can also be classified as regions of low sequence information content or low complexity. Complexity is measured as the amount of amino acid variation found within a length of sequence and this is usually found by a sliding window method that counts the diversity of amino acid content [25].

One key feature of the SAAR distribution identified by previous work is the unusual distribution of asparagine and glutamine repeats. Repeats of these amino acids are associated with neurodegenerative diseases in humans and for this reason they are often annotated as prion-like domains [22]. *P.falciparum* has been found to contain an exceptionally large number of prion-like domains (over 1300 which is about one quarter of the total number of proteins).

The starting point for the study of Pizzi and Frontali was the observation that many malarial proteins are longer than their homologues in other species and that these insertions corresponded to regions of low complexity [23]. They analyzed the distribution of low complexity regions in chromosome 2 and 3 of *P.falciparum* and they found that in most cases this low complexity was associated with hydrophilicity, and in particular with asparagines.

Pizzi and Frontali assumed that these low complexity insertions would form non-globular domains extruded from the core of the protein so that they would not impair the correct folding and functioning of the protein [23]. This view is in disagreement with the assumed prion-like nature of asparagine and glutamine containing low-complexity regions, of Singh *et al*. [22]. Experimental evidence has found that proteins containing glutamine tracts are recruited into amyloid plaque formation after plaque nucleation by molecules of the disease causing prion-protein [26]. Given their chemical similarity the same is likely to occur with asparagine repeats, which have been shown to be rare within mammalian genomes [27]. However this behavior might be limited to repeats of only these two amino acids. Long SAARs can be tolerated in bacteria and yeast, and it might be that the classification of these low complexity regions as prion domains is misleading. In the absence of crystal structures for the proteins containing the long insertions, we need to find other evidence for the evolution and function of these inserted regions by comparing the malarial proteomes.

Intrinsically unordered regions and low complexity regions are ambiguous terms because they depend on the definition and algorithmic determination of these regions, and different groups have taken different approaches. The definition of a SAAR is much less ambiguous which makes them ideal for comparative studies. Now that complete proteomic data is available for six primate and rodent species of malaria it is possible to take a systematic across species approach to understanding the evolution of SAARs.

### The null hypothesis

The key question in identifying patterns in biological sequence data is distinguishing significant patterns that indicate evolutionary relationships or the effects of selection from artifacts, patterns that arise merely by chance. In the case of SAARs the null hypothesis assumes that the SAAR distribution has arisen randomly, rather than under natural selection. A number of different models are available for modeling the expected SAAR distribution under the null hypothesis.

The simplest null model (Model A) is that based on the amino acid propensity this assumes that repeats occur randomly [18], [28]. The probability of a run of amino acids arising by chance can be calculated from the amino acid frequencies for a particular protein. The probability of a run of *n* residues of an amino acid which occurs with probability *f* within a protein is;
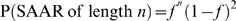



The final term takes into account that the amino acids adjacent to the repeat must be different to those in the repeat, so that the count of repeats of length n does not include repeats of length >n. This has to be multiplied by the number of different starting points for the SAAR. To give an expected number of repeats, of length *n*, of a specific amino acid within a protein of;
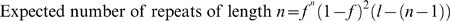



This can then be summed over the whole proteome to give the total numbers of expected repeats.

A second model (Model B) is the ball and urn model proposed by Karlin and co-workers which describes the fundamental word-length of a genome or proteome, this method is known as r-Scan analysis [29], [30]. This does not describe the SAAR distribution itself, but it does define a limit to the word size beyond which you would not expect to see more than one example of a particular word within the sequence. Where there are a large number of SAARs beyond that threshold length this indicates that these repeats are significant words and are likely to be under selection.

A final alternative model (Model C) for the null hypothesis is to use a Markov model approach and assume that the probability of the next amino acid in the sequence (n) is only dependent on a particular preceding amino acid [31]. The correlated amino acid can be at position n-1, n-2, n-3 and so on determining the order of the Markov process. This is a powerful technique in DNA where CpG (C adjacent to G in the sequence) occurs less frequently than expected outside of coding regions. In protein sequences there will be similar effects within secondary structural elements with n-3 or n-4 relationships in helices and n-1 relationships in beta strands. In the case of SAARs these are low complexity regions where there is an absence of secondary structure and where local correlations are unlikely to be related to features in the protein, and so models were only calculated for n, n-1 and n-2, that is a zeroth, first and second order Markov process respectively.

## Results

The SAAR distributions for the available *Plasmodium* proteomes were calculated using Perl scripts written by the author for all of the amino acids. These are the same scripts used to generate the COPASAAR database [18]. The SAAR distribution for *Dictyostelium discoideum* was also calculated as a control for the effect of AT content, as the *D.discoideum* genome has a similar AT content to *P.falciparum* within the coding regions. The complete table of SAAR data is available as supplementary table S1.

This table includes the number of repeats of length 2 to 20 and over 20 residues in length for each amino acid in each proteome, the total number of residues for each amino acid in the proteome, the number of residues for each amino acid contained within repeats, the number of residues for each amino acid present as a “singlet”. This data was then used to calculate a series of proportions. These are;

The **amino acid propensity**, this is the number of amino acids of certain type divided by the total number of amino acids in the proteome.

The **repeat propensity**, this is the number of residues of a particular amino acid that are found in repeats divided by the total number of residues of that amino acid in the proteome.

The **singlet propensity**, this is the number of residues of an amino acid that are found in isolation divided by the total count of that amino acid in the proteome.

The **repeat proportion**, this is the number of residues of an amino acid that are found in repeats divided by the total number of amino acids found in repeats for a proteome.

The **singlet proportion**, this is the number of residues of an amino acid that are found singly divided by the total number of isolated amino acids in the proteome.

The **repeat residual**, this is defined as:
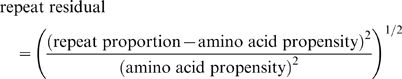



The **singlet residual** is defined as:
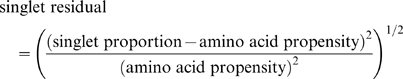



If the proteome sequence was homogeneous then there would be the same proportion of residues for an amino acid in the repeat regions as are found in singlets and this would be the amino acid propensity. Deviations from homogeneity indicate selection for or against repeat regions.

**Figure 1:**
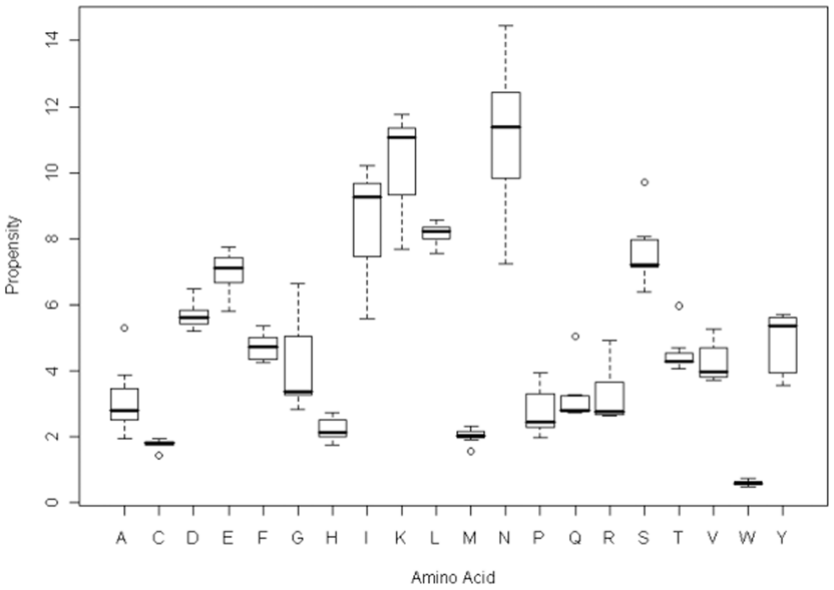
A boxplot of the amino acid propensities across the seven species in the study.

The amino acid propensity plots for all of the organisms are shown in figure 1. The boxplots show the 1^st^ and 3^rd^ quartiles and the bold line indicates the median, with the whiskers indicating the interquartile range. Any values that are over 1.5 times the interquartile range above the 3^rd^ quartile or below the first quartile are marked as **outliers** with circles. The outliers for the boxplots are summarized in table 1.

**Table 1:** A summary of the outliers from the boxplots in figures 1 to [Fig pone-0006231-g002] [Fig pone-0006231-g003] [Fig pone-0006231-g004] [Fig pone-0006231-g005] 6 and 8 to [Fig pone-0006231-g009] 10.

Boxplot	*P.berghei*	*P.chabaudi*	*P.falciparum*	*P.knowlesi*	*P.vivax*	*P.yoelii*	*D.discoideum*
Amino Acid Propensities					A		M, S, T
Amino Acid Propensities in Repeats			D		A, L	L	F, Q, S, T
Proportions of Amino Acids in Repeats and Singlets					A		Q, T, L,S (singlet) K(repeat)
Repeat Residuals					A		F, K, S, T
Singlet Residuals					A		F, K, W
Repeat Proportions Across Species	N, K	N, K	N, K			N, K	N
z-scores for zeroth order Markov model	K		N	K	K, E	Q	
z-scores for first order Markov model			N	N, D, K			
z-scores for the second order Markov model		K	N, K	K, D	D, E, K		

The boxplots show that there is considerable variation in the amino acid propensities between species for glycine (G), isoleucine (I), lysine (K) and particularly asparagine (N). There is almost no variation in the rare amino acids, cysteine (C), methionine (M) and tryptophan (W) and leucine (L) shows an unusually small variation across species. The rare amino acids occur in such small numbers that repeats do not make a significant contribution to the repeat distributions in some organisms.

The outlier for alanine (A) is *P.vivax*, for methionine is *D.discoideum*, for glutamine (Q) is *D.discoideum* for serine (S) is *D.discoideum*, and for threonine (T) is *D.discoideum*. That only a single *Plasmodium* species can be identified as an outlier from the amino acid propensities shows that they form a tightly clustered group, which have few distinguishing features at the level of amino acid composition.

**Figure 2 pone-0006231-g002:**
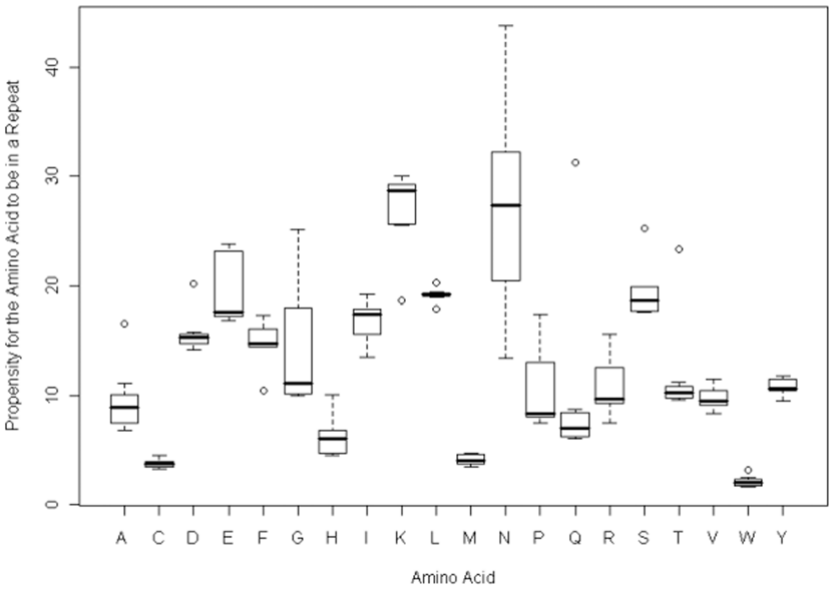
A boxplot of the amino acid propensities for being found in a simple amino acid repeat across the seven species.

Figure 2 shows the propensity for each of the amino acids to be in a repeat across all the species in the study. The variation in isoleucine, lysine and tyrosine repeat propensities are smaller than those seen for the proteome wide propensities, whereas for asparagine and proline (P) show a larger variation. The outliers are *P.vivax* for alanine, *P.falciparum* for aspartic acid (D), *D.discoideum* for phenylalanine (F), *P.vivax* and *P.yoelii* for leucine (*P.vivax* is over-represented, and *P.yoelii* under-represented), *D.discoideum* for glutamine, serine and threonine.

*P.vivax* was an outlier in the alanine content of its proteome and so it is not surprising that alanine is also over-represented in repeat regions of this species.

**Figure 3 pone-0006231-g003:**
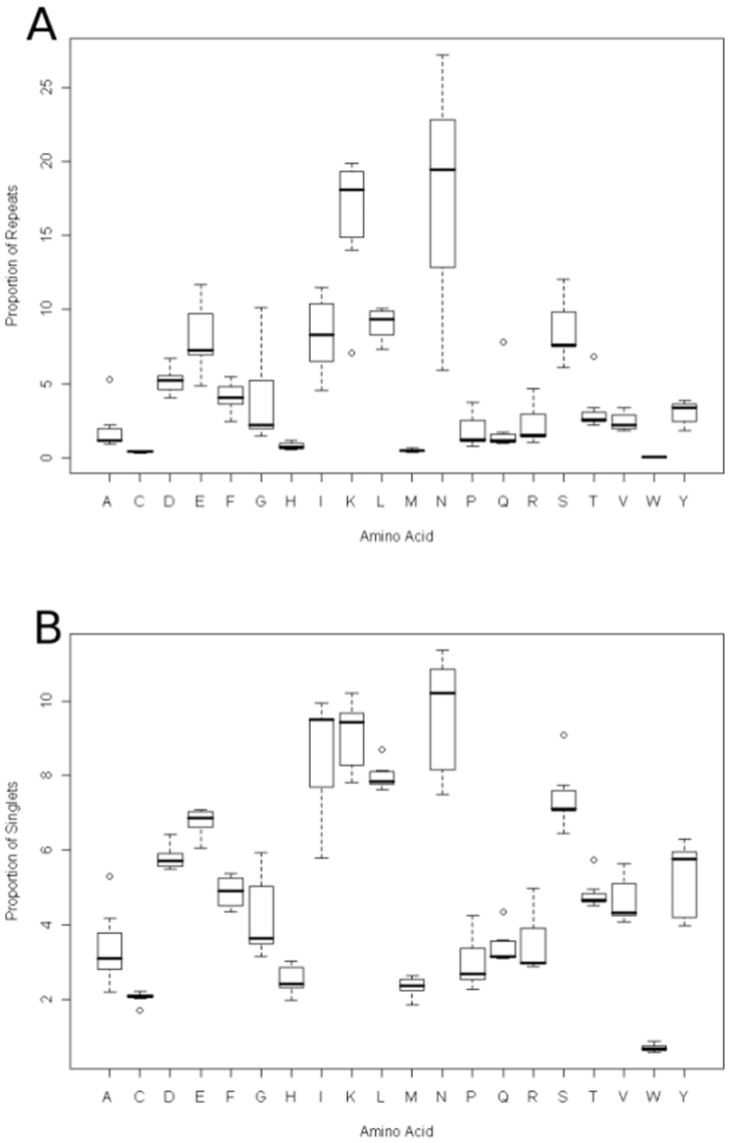
A boxplot of the proportions of amino acid residues found in repeats (3a) and as singlets (3b) across the seven species.

**Figure 4 pone-0006231-g004:**
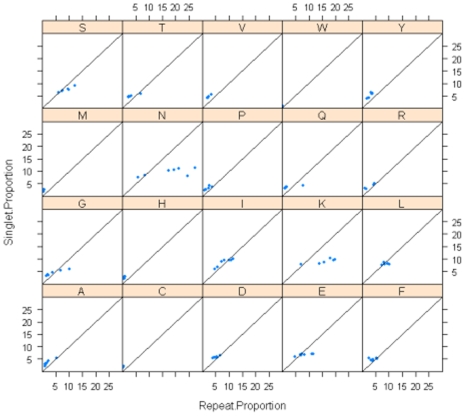
Scatterplots of the singlet and repeat proportions for each of the amino acids. Deviations from the diagonal indicate a preference for the amino acid being in either singlets or repeats.

Figures 3a and 3b show the proportions of repeats and singlets for each of the amino acids. The most frequent amino acids will contribute the most to both repeats and singlets but any differences between the two plots indicate a preference for being either in a singlet or repeat. The distributions for both lysine and asparagine are very different between repeats and singlets, with both contributing to a much higher number of repeats. There is a considerable spread for asparagines in the proportion of repeats but only a much smaller variability in singlets. The outliers in the proportion of repeats plots are *P.vivax* for alanine and *D.discoideum* for lysine, glutamine and threonine. The outliers for the proportion of singlets plot are, *P.vivax* for alanine, and *D.discoideum* for leucine, glutamine, serine and threonine. Figure 4 shows the scatterplots between the singlet and repeat proportions, deviations from the diagonal show which amino acids exhibit a preference for either being found as a singlet or as a repeat. Asparagine, isoleucine and glutamic acid show the largest deviations and all show a preference for being in repeats over singlets.

**Figure 5 pone-0006231-g005:**
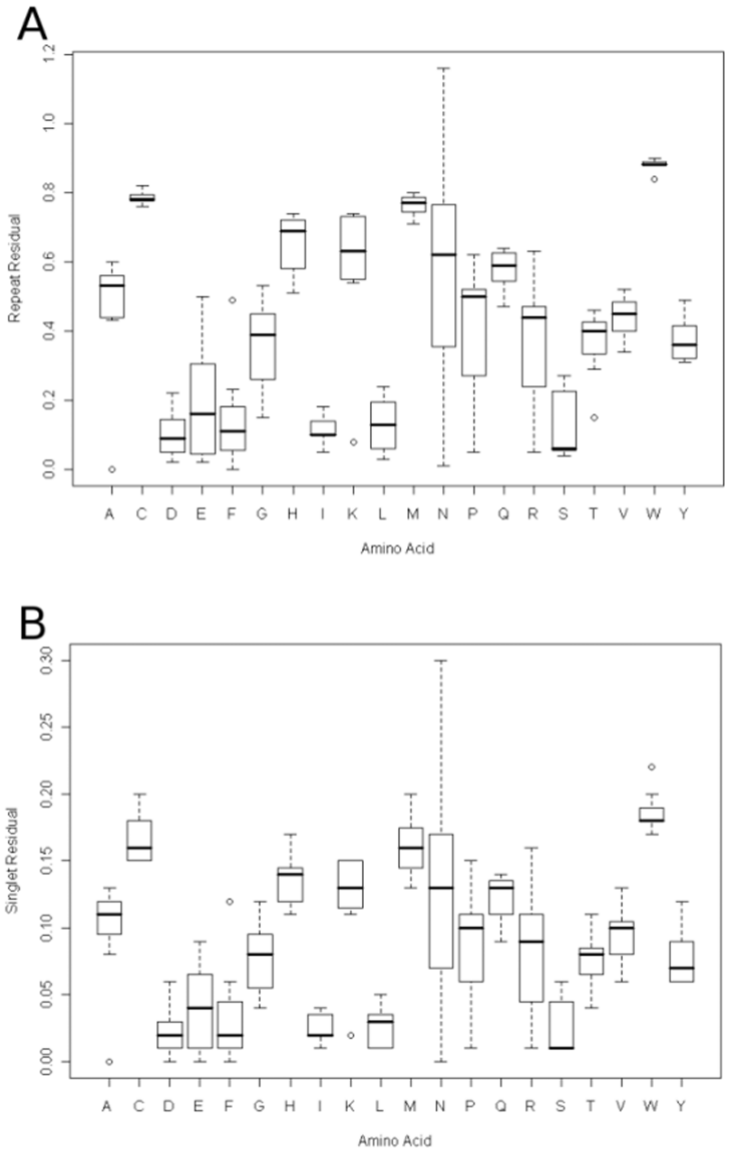
A boxplot of the repeat (4a) and singlet (4b) residuals as defined in the results section of the paper.

Figures 5a and 5b show the repeat and singlet residuals, these are an absolute measure of the deviation of the number of residues in repeats and singlets from that expected from the amino acid propensity in the proteome. A residual of zero shows that there is no preference for singlet or repeat stretches. The singlet residuals are much smaller than those of the repeats because of the much larger numbers of residues in singlets compared to repeats smoothing out the variation from the proteome average. Asparagine has the biggest variation across species for both residuals covering the entire range, proline, arginine and glutamic acid also show a wide variation. Most amino acids have residuals above zero indicating some preference for either repeats or singlets. The most pronounced of these is tryptophan which has a very tight distribution between species at a high residual. The amino acids with residuals close to zero are, aspartic and glutamic acids, phenylalanine, isoleucine, leucine and serine.

The outliers for the repeat residual plot are *P.vivax* for alanine, and *D.discoideum* for phenylalanine, lysine, serine and threonine. The outliers for the singlet residuals are *P.vivax* for alanine and *D.discoideum* for phenylalanine, lysine and tryptophan. This confirms that the alanine distribution of *P.vivax* is unusual, but also shows that it is not caused by the abundance of alanine repeats but rather that alanine is in equilibrium between repeats and singlets.

**Figure 6:**
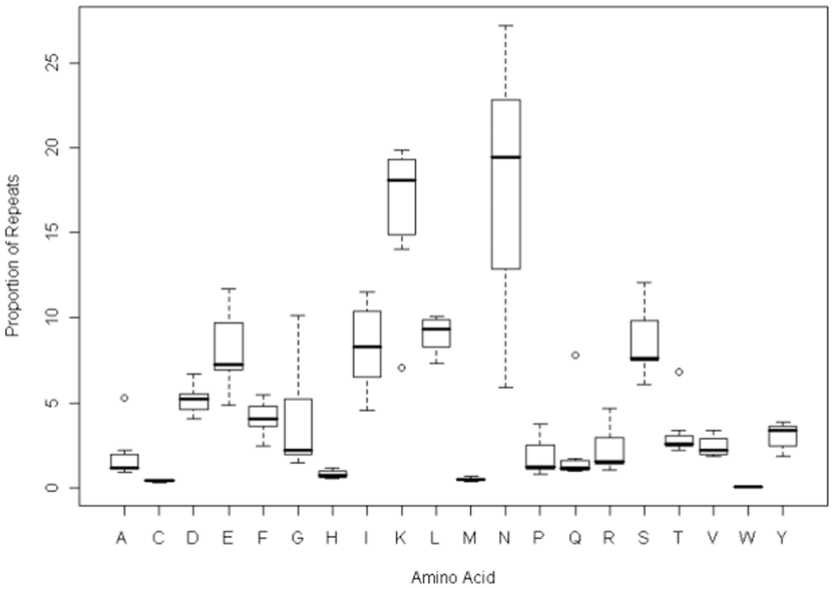
A boxplot comparing the repeat proportions across all the amino acids between species.

Figure 6 shows a species by species comparison of the repeat proportions for all of the amino acids. This shows if any amino acid is particularly under or over represented in the repeats for a particular species. In the cases of *P.knowlesi* and *P.vivax* none of the amino acids has an exceptional repeat proportion, but in the other malarial species two amino acids are outliers and in *D.discoideum* one. The outlying amino acids for the four malarial species are lysine and asparagine, and the outlier for *D.discoideum* is asparagine.

One interesting feature from the plot is the distance between the lysine and asparagines repeat proportions. In *P.berghei* they superpose and in *P.yoelii* and *P.chabaudi* they are very close while in *P.falciparum* there is a more extreme asparagine repeat proportion. This clustering is in agreement with the *Plasmodium* phylogenetic analysis which suggests that the rodent malarial species *P.berghei, P.yoelii* and *P.chabaudi* form one cluster, *P.falciparum* forms another on its own and then more distantly there is a cluster containing *P.knowlesi* and *P.vivax* [*7*].

**Figure 7:**
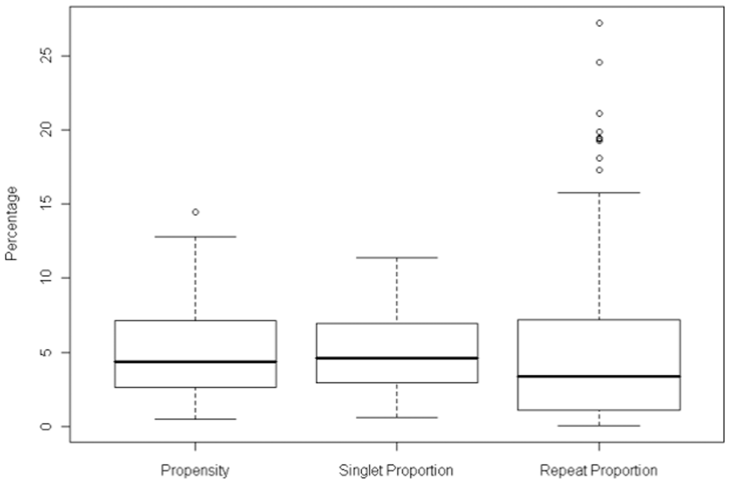
A boxplot comparing the variation between the amino acid propensities and their proportions in repeat and singlets.

The final summary plot of the data is given in figure 7 and shows the spread of the amino acid propensities against the spread of the singlet and repeat proportions. All have to have the same mean of 5% (the mean percentages of 20 amino acids), but there is a much greater spread with a significant number of outliers in the case of the repeat proportions. The outlier for the amino acid propensity is asparagine in *P.falciparum*. The outliers for the repeats proportions are asparagine in *P.yoelii, P.falciparum, P.chabaudi, P.berghei* and *D.discoideum*, and lysine in *P.yoelii P.knowlesi*, *P.falciparum, P.chabaudi*, and *P.berghei*.

Thus far the results presented have been based only on the summary statistics of the SAAR distributions rather than on the SAAR distributions themselves. Three different models have been used to test for the significance of features within the SAAR distribution.

### Model A

The complete expected SAAR distributions for all of the malarial parasites and *D.discoideum* are given in supplementary table S2. The expected distributions of repeats take into account the amino acid frequencies for each protein and so they are still not at equilibrium, where the amino acid frequency in the repeat regions will be the same as that for the proteome. The same summary plots that were given for the observed distribution are given in supplementary figures S1, S2, S3, S4. *D.discoideum* is once again frequently the outlier in the plots. Sometimes the variation between species has been reduced for a particular amino acid and this can result in fewer outliers and a higher interquartile range, or sometimes new outliers occurring where the interquartile range has become smaller.

The observed and expected SAAR distributions can be compared directly on a repeat by repeat basis. Most of the expected SAAR distributions decay much faster than the observed distributions, and in general the rate of decay of the frequencies of repeats is so fast and there is such a long tail that graphical comparison is difficult. The presence of zero frequencies makes it impossible to use logarithmic scales for plots for repeat lengths where either the observed or expected numbers of repeats are zero.

For bins where the expected counts are greater than 5 the chi-squared test can be used to evaluate the difference between the two distributions, but for all amino acids these rapidly go to zero (see table 2 for the chi-squared test values for *P.falciparum*). The results indicate that in this case the repeat distributions are significantly different from those expected from the amino acid distributions alone.

**Table 2:** The chi-squared test values for the observed against the expected SAAR frequencies in *P.falciparum* under null hypothesis model A.

Amino Acid	Chi-squared	Degrees of Freedom	P	Log(p)
Asparagine	102404	6	0	−51181
Alanine	511.85	2	7.1×10^−112^	−255.9
Cysteine	47.72	1	4.9×10^−12^	−26.04
Aspartic Acid	7926	3	0	−3958
Glutamic Acid	3719	5	0	−1848
Phenylalanine	3241	3	0	−1616
Glycine	641.4	2	5.2×10^−140^	−320.7
Histidine	503.4	2	4.7×10^−110^	−251.73
Lysine	40911	5	0	−20440
Isoleucine	329.3	3	4.5×10^−71^	−161.9
Leucine	1154	3	7.0×10^−250^	−573.7
Methionine	82.58	1	1.0×10^−19^	−43.73
Proline	434.5	2	4.5×10^−95^	−217.25
Glutamine	286.1	2	7.5×10^−63^	−143.05
Arginine	1896	2	0	−948
Serine	4671	3	0	−2331
Threonine	425.0	2	5.1×10^−93^	−212.5
Valine	78.55	3	8.7×10^−18^	−37.30
Tryptophan	78.29	0	NA	NA
Tyrosine	238.5	3	1.6×10^−52^	−116.73

### Model B

In the r-scan model the formulae for calculating the natural word length is given by:
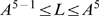



Where A is the length of the alphabet (for amino acids A = 20), L is the length of the sequence in which the words can be found (the proteome length) and *s* is the natural word length [30].

This mathematical model shows that if there are frequent numbers of SAARs longer than the natural word length then this is an unusual distribution of words and they are likely to be significant. This implies that they will be functionally important and under selection. If words of six letters are taken as the threshold across *Plasmodia* then it is clear that repeats are over-represented for nearly all of the amino acids. Only cysteine, and tryptophan do not have SAAR distributions with repeat lengths of six amino acids or longer in all species.

Alanine, glycine, histidine, isoleucine, leucine, methionine, proline, arginine, threonine and valine have low numbers of repeats longer that this threshold, but there is some variation between species. So for example *P.vivax* has a longer tailed distribution for alanine SAARs than any of the other species. Within the malarial species *P.yoelii*, *P.chabaudi* and *P.berghei* have more isoleucine repeats than *P.falciparum*, *P.knowlesi* and *P.vivax*. Within *Plasmodia* glutamine has longer repeats in *P.falciparum* and *P.vivax*, which does not agree with the phylogenetic clustering found previously, as the repeats are absent from the *P.knowlesi* proteome, but this would agree with a host specific adaptation as both are human parasites.

There are very significant long tailed distributions across all species for aspartic and glutamic acids, phenylalanine, lysine, asparagine and serine. With the exception of phenylalanine these are all hydrophilic amino acids that are usually found on the surface of proteins, which are regions more amenable to insertions and deletions.

### Model C

The zeroth, first and second order Markov models were constructed for a word length of four using the R'MES program [32]. For the purposes of this study we are only interested in the unusual frequencies of homogeneous words (AAAA, CCCC etc).

The complete tabulated data from the Markov models is given in supplementary table S3. The results are given as z-scores which are a test against the assumed Gaussian distribution of the words. High z-scores correspond to low probabilities of this occurring by chance and negative scores indicate an under-representation to that expected. A z-score of ∼70 corresponds to a p value (a probability of this word frequency arising by chance) of 10^−323^ and scores of 10 correspond to a p value of 10^−26^.So any values above 10 can be considered significant [32].

Over long sequence lengths such as a proteome the Gaussian approximation is appropriate as the frequencies are expected to be large for most of the words. This will break down in the case of cysteine, methionine and tryptophan and so the results for these residues should be treated with caution.

The data for *D.discoideum* shows that repeats of almost all amino acids occur at a significant rate compared to the random distribution of amino acids for all three Markov models and so it was excluded from any further analysis and comparison to the malarial models.

**Figure 8:**
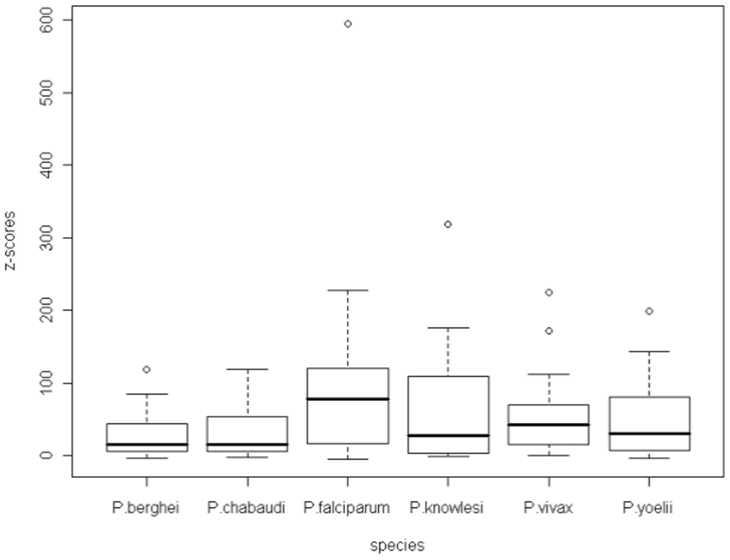
A boxplot of the z-scores for the homo-repeat words in the zeroth order Markov model across all of the malarial species.

Figure 8 shows the z-scores for the zeroth order Markov model for all of the malarial species. There is a very wide range of z-score with only isoleucine having a negative values indicating it occurs with lower frequency than expected. Variations between the species are similar, except that *P.falciparum* has a larger interquartile range. The outliers are lysine and glutamic acid for *P.vivax*, lysine for *P.knowlesi* and *P.berghei*, glutamine for *P.yoelii*, and asparagine for *P.falciparum*.

**Figure 9 pone-0006231-g009:**
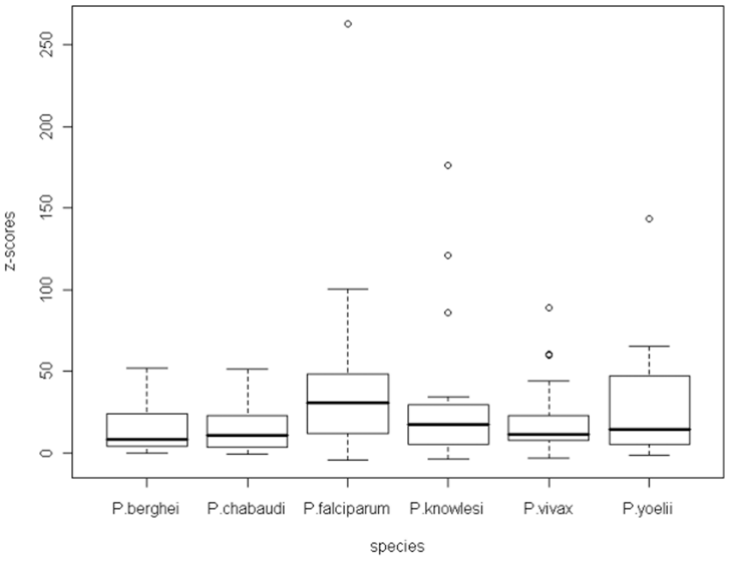
A boxplot of the z-scores for the homo-repeat words in the first order Markov model across all of the malarial species.

Figure 9 shows the z-scores for the first order Markov model. These are much lower than for the zeroth order model as the model will fit the data better as the length of sequence which is used to predict the probabilities increases. In this case the outliers are asparagine for *P.falciparum*, asparagine, aspartic acid and lysine for *P.knowlesi*, lysine, aspartic and glutamic acids for *P.vivax* and glutamine for *P.yoelii*.

**Figure 10:**
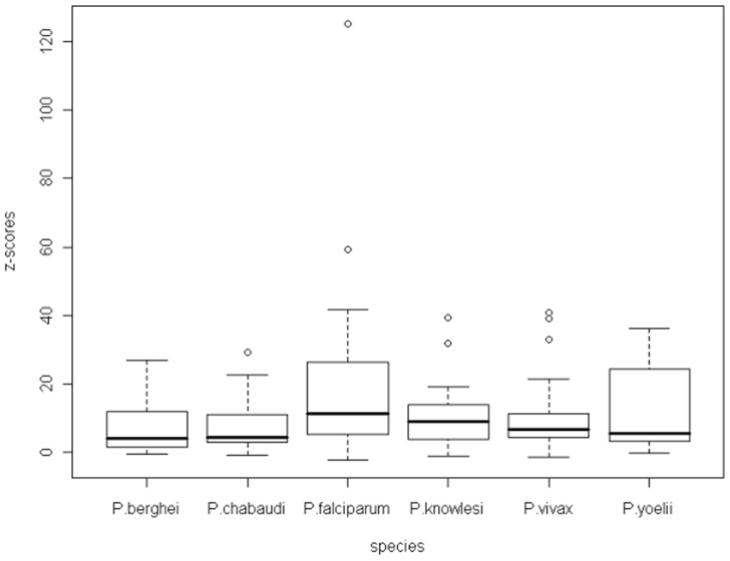
A boxplot of the z-scores for the homo-repeat words in the second order Markov model across all of the malarial species.

Figure 10 shows the z-scores for the second order Markov model. The variation in z-score is now considerably lower than for the zeroth and first order models. The outliers in this case are lysine for *P.chabaudi*, asparagine and lysine for *P.falciparum*, lysine and aspartic acid for *P.knowlesi*, and lysine, aspartic and glutamic acids for *P.vivax*.

## Discussion

There have been a number of previous studies that have examined the occurrence of low complexity regions or intrinsically unordered regions in eukaryotes including *P.falciparum* *[9], [21]–[23]*. Low complexity regions are defined by using information theoretical measures and these regions overlap with SAARs [25]. The overlap between SAARs and intrinsically disordered regions is less clear as there are a number of methods for calculating IURs and they have been shown to occur in complex sequence regions [21]. The very unusual distribution of asparagine repeats in *P.falciparum* has been a particular focus of attention because of the association of these repeats with prion like domains [22].

In this study the comparisons were restricted to the *Plasmodia* with *D.discoideum* added as a control for high A–T content. This is the first time that a systematic comparison of the SAAR distributions has been possible for all of the rodent and primate malarial species across their entire proteomes. By using only a series of closely related species that have similar lifestyles and selective pressures this should reduce the number of variables that have to be considered in proteome evolution. Figure 1 showed that *D.discoideum* is clearly an outlier in its SAAR distribution, which seems to reflect a runaway accumulation of repeats, whereas most of the malarial species do indeed share similar SAAR distributions. The distinctive nature of the SAAR distribution in *D.discoideum* is further highlighted in figures 2–5, which show that it is frequently the outlying species when repeats are examined on an amino acid by amino acid basis. This suggests that SAAR distribution could be used to group related species. Although this finding is not sufficient to determine that the differences provide a robust metric for comparing distances between them.

The SAAR distributions are a simple sequence feature that potentially can be used in proteome comparison. The results show *P.knowlesi* and *P.vivax* which have A–T contents of around 60%, form a distinct group based on their use of hydrophobic amino acids in repeats whilst the higher A–T content malarial parasites (*P.berghei, P.chabaudi*, *P.falciparum*, and *P.yoelii*) group together in their amino acid preferences in SAARs with outliers for asparagine and lysine. This effect of A–T content had been reported previously, and this clustering could have been achieved based on A–T content alone. A detailed examination of the SAAR distributions from Figure 6 shows that *P.falciparum* is an extreme outlier for asparagine and that the rodent species share a similar repeat proportion.

There has been extensive discussion in the literature as to whether the SAAR distribution of asparagine results from the A–T content alone [9], [22], [23]. The difference in A–T content between the rodent high A–T content group (*P.berghei*, *P.chabaudi*, and *P.yoelii*) and *P.falciparum* is 2% but there is a large difference in repeat distribution for lysine and asparagine repeats between the two groups. Analysis of proteins from *P.reichenowi* has shown a similar distribution of long asparagine repeats to that found in *P.falciparum* and so it is unlikely to be an artifact from sequencing and gene identification [9]. A–T content is certainly playing a part in the number of lysine and asparagine repeats but the absence of long asparagine repeats and preference for lysine repeats found in the rodent species suggests that these repeats are under some selective determination which is lineage specific and which evolved after the rodent and primate malaria species diverged around 100 million years ago.

### Expansion Mechanism

There are two possible mechanisms for repeat expansion, polymerase slippage events and recombination [33], [34]. If polymerase slippage is the dominant force then we would expect to see a high degree of variability in repeat lengths and homogeneity between phylogenetic analysis carried out between sequence regions upstream and downstream of repeats [9], [35].

Recombination has been proposed as an expansion mechanism for producing long-tailed repeat distributions i.e. SAAR distributions where there are a low number of very long repeats [36], [37]. There is always the chance that a very long repeat might arise sporadically such as the single very long lysine repeat in *Arabadopsis thaliana* but where the SAAR distribution contains a very high number of short repeats and a small number of many long repeats this can be described by a power-law. Power laws arise frequently in physics and statistical physics has been used to model repeat expansions to show how these distributions can be obtained [33]. The underlying assumption of these models has been that expansion and contraction of repeats is through recombination.

In the case of tri-nucleotide repeat expansion associated with human disease polymerase slippage is the most likely contributor to repeat expansion [13]–[15]. However these studies do show that there are differences between species as the repeat expansions have different critical lengths in mouse and humans, and the rates of perfect repeat loss because of point mutations also varies significantly between species, so that in some species repeat shortening and loss is more frequent than expansion [12], [16].

The variability of asparagine repeat lengths in *P.falciparum* isolates suggests that polymerase slippage is the most frequent cause of expansion of these repeats. However, given the high recombination rate of *P.falciparum* and the increased likelihood of recombination break-points occurring in repetitive DNA sequence motifs this is not always the case [38]. De Pristo *et al.* have shown that recombination plays a part in at least some repeat expansions by examining the phylogenies of flanking sequences either side of the repeat region [9].

If the SAAR distributions were solely dependent on repeat expansion mechanisms then the number of repeats observed would only depend on the codon frequencies in the genome. In at A–T rich genome we would expect lysine, asparagine, phenylalanine and isoleucine to have similar repeat distributions but it is clear from the analysis that they do not (figure 3a and supplementary table S1). The repeat distribution for isoleucine tails off very quickly and phenylalanine is under-represented amongst the proportion of repeats. The variability in the repeat distributions between lineages (supplementary table S1) indicates that repeat expansion is not a stochastic process and that selection plays a role in some amino acid repeat distributions.

### Selection and Neutrality of Repeats

Variations between the SAAR distributions of closely related species with similar genome content suggest that selection plays a part in producing the observed data. Where orthologous genes do not contain repeat regions it has been assumed that the inserted repeats are selectively neutral mutations that do not undergo purifying selection [39]–[41]. The other possibility is that repeat regions might have acquired a function. In the case of *P.falciparum* long asparagine repeats have been identified in antigenic proteins and so a number of studies have suggested that these repeats play a part in the evasion of the host's immune response [21], [42].

Recent results have shown that whilst many SAARs are more commonly found in regions with a high degree of sequence polymorphism an unexpected number of repeats were present in regions of low variation [35]. These were identified as ancient repeats that often have orthologues in other species, and which can be inferred to be retained by selection as functionally important.

In the case of human tri-nucleotide repeat diseases these are often dominant homozygous diseases which are caused by a gain of protein function [14], [26]. The expansion of repeats causes the proteins to become involved in additional protein-protein interactions, which can be of relatively low specificity resulting in protein aggregation. It is for thus reason that long stretches of glutamine and asparagine repeats have been identified as prion domains.

The very large number of asparagine repeats in *P.falciparum* show that within this species selection does not act strongly against on these repeats regardless of their potential prion like nature. In *P.falciparum* asparagine repeats are found in a wide variety of proteins. What is most unusual is that they are found in protein globular domains that are usually resistant to repeat insertion [21]–[23]. However they are mostly found in sequence regions that have a high degree of sequence variation and that have been identified as probable surface loops that are likely to form extruded unfolded domains. There is limited experimental evidence that suggests asparagine repeats play a functional role. As mentioned above a long asparagine repeat was first identified in an antigen protein, but they may also play a role in catalysis as several enzymes containing long repeats have been characterized [43], [44]. Asparagine repeats in *P.falciparum* occur in far greater number and over far greater lengths than in any other malarial species, the next largest numbers of repeats are found in *P.berghei* which has less than half the number of asparagine residues in repeat regions.

*P.falciparum* also contains a number of glutamine repeats although *P.vivax* contains more residues and *P.knowlesi* slightly fewer, while the rodent malarial species have far fewer. While all three of these species are primate malarias *P.falciparum* and *P.vivax* are human malarias and *P.knowlesi* infects long-tailed macaques and has only very rarely been transmitted to humans. This might suggest a relationship between the asparagine repeat distribution and the parasite host, and that glutamine repeats are adaptive to primate parasites. This is a reasonable finding given the preference for glutamine repeats over asparagine repeats in humans and the large number of glutamine repeats in the human proteome.

One other factor that needs to be taken into account is the number of unique proteins in the proteome as this could make a significant contribution to the differences in repeat distributions between the species. Currently the *P.falciparum* genome is being re-annotated and the *P.berghei* and *P.chabaudi* genomes are not well characterized and contain a larger number of protein coding sequences than are likely to be present in the final proteome. Current estimates based on comparisons with *P.falciparum*, *P.knowlesi*, and *P.vivax* suggest that most proteins contain orthologues in the other species and that only between 100 and 200 proteins out of a total proteome of 5000 proteins will be unique to a given species [45].

### Model A

The chi-squared test results for the SAAR distributions from *P.*falciparum are given in table 1 and show that the repeat distributions differ significantly from the null hypothesis for all of the amino acids. Even methionine, cysteine, leucine and isoleucine show significant deviations from their expected values. This reflects the different mutational processes and rates of diversification associated with single amino acid changes and repeat proliferation. Repeat expansion is a much more rapid process than point mutation and it has been associated with phenotypical and morphological changes.

The performance of the model is so poor, that clearly the null hypothesis has to be rejected for *P.falciparum* but an interesting question is if this is always the case. The chi-squared test for asparagine in the most extreme and so a further question would be if the null hypothesis for asparagines is rejected for all eukaryotes. Table 3 shows the chi-squared tests for the asparagine repeat distributions across a number of eukaryotic organisms. These results show that model A is valid in the case of *Paramecium tetraurelia*, and that it would only be rejected at the 5% level in *Mus musculus*. This variation between species and between amino acids in the chi-squared values shows that there is considerable variation in the mechanisms of repeat expansion and selection between eukaryotes and suggest that lineage specific distributions of SAARs might be a general feature of eukaryotic evolution.

**Table 3:** The chi-squared test values for the observed against the expected number of asparagine repeats in eukaryotes under null hypothesis model A.

Species	Chi-squared	Degrees of Freedom	P	Log(p)
*P. falciparum*	102404	6	0	−51181
*P. berghei*	660.06	5	2.2×10^−140^	−321.6
*P. yoelii*	2154	5	0	−1067
*P. chabaudi*	168.73	5	1.36×10^−34^	−78.00
*Caenorhabditis elegans*	464.05	4	4.0×10^−99^	−226.5
*Arabidopsis thaliana*	5218	3	0	−2605
*Saccharomyces cerevisaie*	796.09	3	3.0×10^−172^	−394.9
*Mus musculus*	6.92	2	0.031	−3.46
*Homo sapiens*	12.87	2	0.002	−6.44
*Schizosaccharomyces pombe*	24.71	2	4.3×10^−6^	−12.35
*Brachydanio rerio*	63.47	2	1.7×10^−14^	−31.74
*Rattus norvegicus*	30.67	2	2.2×10^−7^	−15.34
*Paramecium tetraurelia*	1.57	2	0.456	−0.785

### Model B

Whilst this model for the significance of the amino acid repeats does help to simplify the comparisons by defining a minimum word length for significance, this obscures the significance of over-abundant short repeats, which are highlighted by the chi-squared analysis of model A. It is interesting to note that the choice of word length agrees with many of the previous studies which only analyzed word lengths above a threshold value [39], [44], [46], [47].

This is a much less detailed approach but one which has been used successfully in previous studies and which in the present study yields useful results for grouping the different malarial species based only on the highly significant repeats. This also reduces the number of amino acids for which the distributions need to be considered as most repeat distributions decay rapidly and do not extend beyond the critical word length.

### Model C

In general the results from the Markov models are comparable with those from model A in identifying the amino acids with significant deviations in the expected number of repeats. As word length is restricted to four this model includes bias in the shorter length repeat distributions. Lysine and asparagine are once more often the outliers but in this case *P.yoelii* rather than *P.falciparum* and *P.knowlesi* are outliers for glutamine. This shows that the significance of features in the amino acid repeat distributions is different between short and long repeats. So organisms which have an over-representation of short repeats might not have a large number of repeats above the critical word length and so these features can be missed when only looking at long repeats.

Surprisingly higher order Markov models are needed to explain repeats, as this was expected to be a feature of secondary structural elements, but this in fact simply reflects the mechanism of repeat expansion. In the case of long repeats they are more likely to get longer still because they have more opportunities for slippage and recombination events to extend them. This highlights the effect that the rich get richer, that will produce a long tailed distribution.

### Conclusions

The results in table 1 show that amino acid repeats are sufficient to distinguish between a series of related species and a more distant one, even if the outgroup shares a similar nucleic acid content to the members of the group. This suggests that SAAR distributions are a potential feature for clustering species. While further work will be required to establish if this is a potential metric that can be used in phylogenetic analysis.

One of the most unexpected results is that for *P.vivax* which has a markedly different distribution of alanine repeats to the other malarial species. Along with *P.knowlesi* this species has a much higher GC content than the other malarial species and they form a lineage that is most divergent from the other species. This lineage specific usage of amino acids is further evidence for differences in selective pressures on repeats between species. More subtle is the findings from the species specific plots shown in figure 6 where asparagine and lysine are outliers for the four high AT content malarial species, but where the distances between he outliers correlates to the existing phylogenetic trees.

As we approach an era of comparative genomics and proteomics we need to find simple features that allow us to calculate phylogenies and evolutionary connections. SAARs provide one possible feature for interspecies comparison and may also shed some light on selection and the evolutionary process.

## Materials and Methods

All of the proteomic data was downloaded as a single fasta file for each organism from the integr8 database at the EBI http://www.ebi.ac.uk/integr8.

Repeat numbers were then analyzed using an in house Perl program that had been developed during the creation of the COPASAAR database of amino acid repeats [18]. Expected repeat numbers were also calculated using in house Perl program also used in the database generation.

The Markov model analysis was carried out using the R'mes program version 3 available from http://migale.jouy.inra.fr/outils/mig/rmes [32].

The data from the Perl programs and the Markov models were tabulated using Microsoft Excel and then all of the subsequent statistical analysis was carried out using R version 2.8.1.

The chi-squared values for tables 1 and 2 were calculated using the formula;
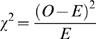



Where O is the observed number of repeats of a given length and given amino acid type and E is the expected number of repeats of the same length and composition. The values were then summed for each amino acid over all repeat lengths that were expected to have a count of 4 or more and compared with the chi-squared distribution with n-1 degrees of freedom, where n is the longest length of a repeat which has a predicted count of more than 4.

## Supporting Information

Table S1 The tabulated SAAR data. Observed simple amino acid repeat distribution.
(0.05 MB XLS)

Table S2 The tabulated expected SAAR distributions calculated using model A.
(0.05 MB XLS)

Table S3 The tabulated z-scores for the Markov models.
(0.03 MB XLS)

Figure S1 A boxplot of the expected amino acid propensities for being found in a simple amino acid repeat under model A across the seven species
(0.03 MB TIF)

Figure S2 A boxplot of the expected proportions of amino acid residues found in repeats (A) and as singlets (B) under model A across the seven species.
(0.04 MB TIF)

Figure S3 A boxplot of the expected repeat (A) and singlet (B) residuals under model A as defined in the results section of the paper.
(0.04 MB TIF)

Figure S4 boxplot comparing the expected repeat proportions assuming model A across all the amino acids between species.
(0.03 MB TIF)
