# Supplementary figures and images for: A Comparative Proteomic Analysis of the Simple Amino Acid Repeat Distributions in Plasmodia Reveals Lineage Specific Amino Acid Selection
Source: PLoS One. 2009 Jul 14;4(7):e6231. doi: 10.1371/journal.pone.0006231 (PMC2705789; doi:10.1371/journal.pone.0006231)

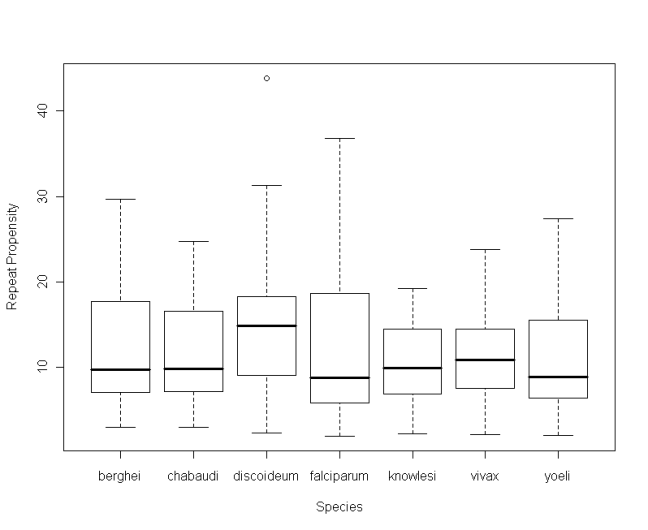

Supplement: Figure S1 — A boxplot of the expected amino acid propensities for being found in a simple amino acid repeat under model A across the seven species (0.03 MB TIF) [file pone.0006231.s004.tif]

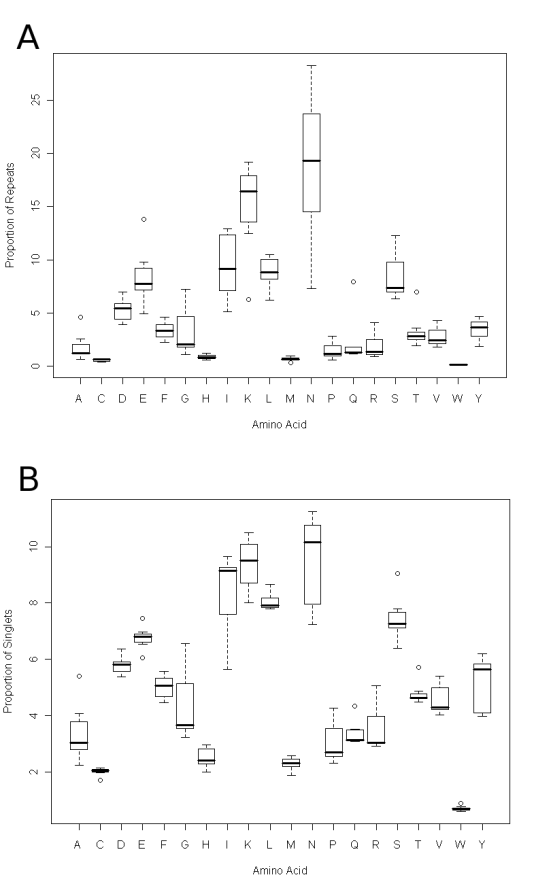

Supplement: Figure S2 — A boxplot of the expected proportions of amino acid residues found in repeats (A) and as singlets (B) under model A across the seven species. (0.04 MB TIF) [file pone.0006231.s005.tif]

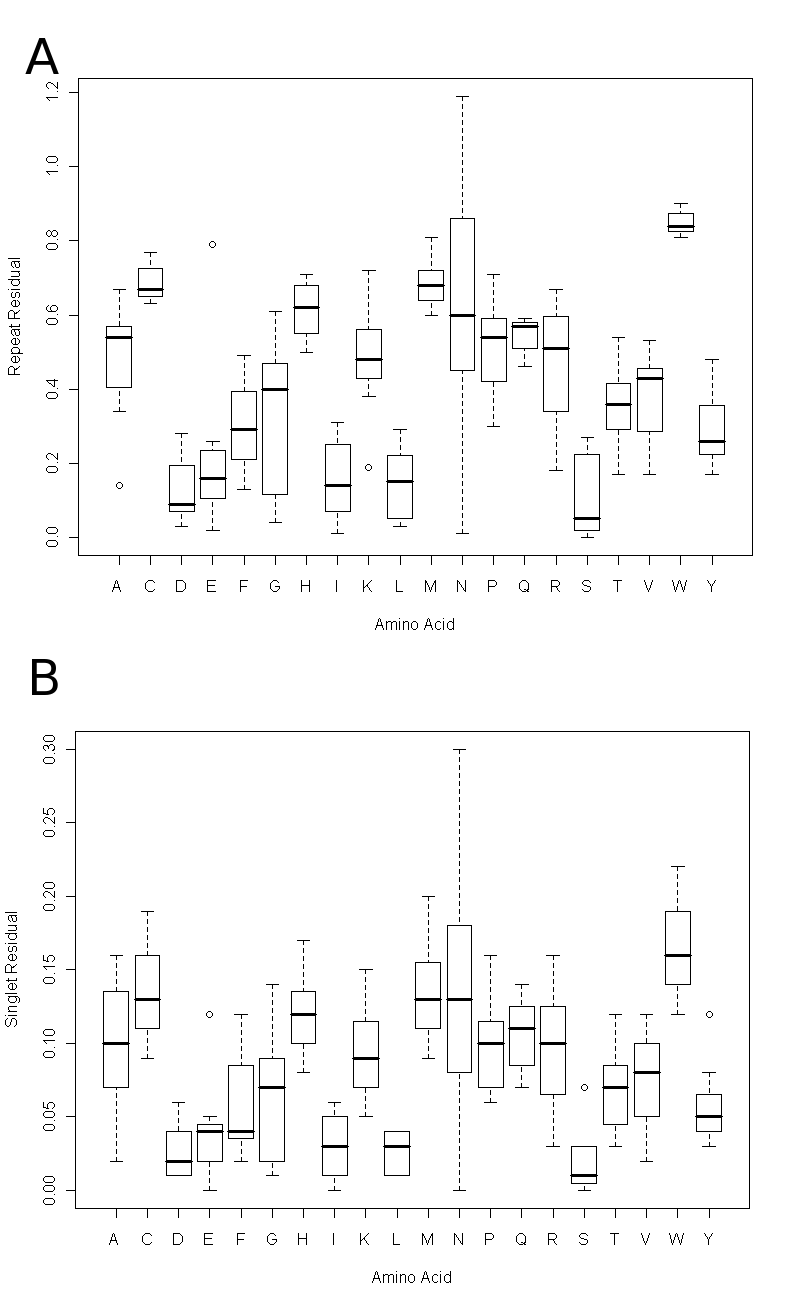

Supplement: Figure S3 — A boxplot of the expected repeat (A) and singlet (B) residuals under model A as defined in the results section of the paper. (0.04 MB TIF) [file pone.0006231.s006.tif]

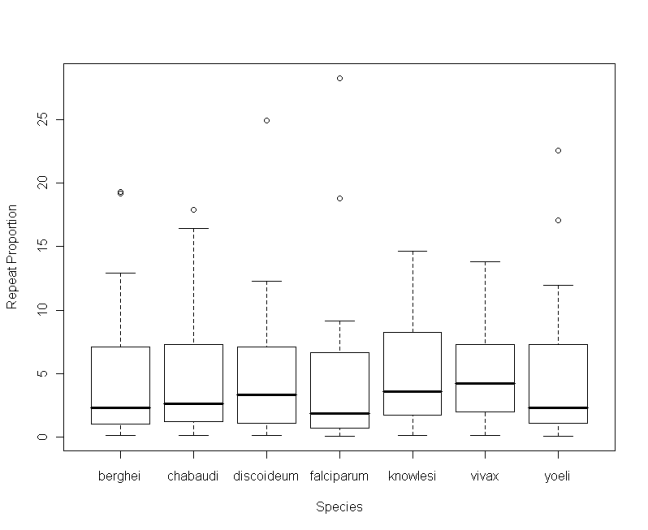

Supplement: Figure S4 — boxplot comparing the expected repeat proportions assuming model A across all the amino acids between species. (0.03 MB TIF) [file pone.0006231.s007.tif]
